# Empathy for Pain: Insula Inactivation and Systemic Treatment With Midazolam Reverses the Hyperalgesia Induced by Cohabitation With a Pair in Chronic Pain Condition

**DOI:** 10.3389/fnbeh.2018.00278

**Published:** 2018-11-16

**Authors:** Caroline R. Zaniboni, Vinícius Pelarin, Daniela Baptista-de-Souza, Azair Canto-de-Souza

**Affiliations:** ^1^Psychobiology Group, Department of Psychology, Center for Education and Human Sciences – Universidade Federal de São Carlos, São Carlos, Brazil; ^2^Graduate Program in Psychology, Universidade Federal de São Carlos, São Carlos, Brazil; ^3^Joint Graduate Program in Physiological Sciences PIPGCF UFSCar, Universidade Estadual Paulista, São Carlos, Brazil; ^4^Institute for Neuroscience and Behavior, Universidade de São Paulo Ribeirão Preto, Ribeirão Preto, Brazil

**Keywords:** social modulation of pain, insula, mice, hypernociception, Benzodiazepine-GABAA system

## Abstract

Empathy for pain is the ability to perceive and understand the pain in the other individual. Recent studies suggested that rodents have this social ability. GABAergic system has receptors in the brain structures involved in emotional processes as well as in the insular cortex. This area has been described as an important key in modulation of pain and empathy. The present study has investigated the role of insula and its Benzodiazepine-GABAA system on social modulation of pain induced by cohabiting with a mouse submitted to sciatic nerve constriction, a neuropathic pain model. The insular cortex function was assessed by the structure inactivation (Experiments 1 and 2); the role of GABA system was evaluated by systemic treatment of midazolam (MDZ 0.5, 1, and 2 mg/kg) (Experiment 3); and the role of GABAA receptors of insula were studied by bilateral MDZ (3 and 30 nmol/0.1 μl) microinjections in the structure (Experiment 4). Male Swiss mice were housed in groups or dyads. On dyads, after 14 days of cohabitation they were divided into two groups: cagemate nerve constriction and cagemate sham (CS). After 14 days of familiarity, cagemates were evaluated on the writhing test. For group-housed, insula inactivation did not change nociception. For dyad-housed, cohabiting with a mouse in chronic pain increased the nociceptive response and the insula inactivation has reverted this response. Systemic MDZ attenuated nociception and intra-insula MDZ did not alter it. Our results suggest that cohabitation with a pair in chronic pain induces hypernociception, insula possibly modulates this response and the GABA system is also possibly involved, but not its insular mechanisms.

## Introduction

The ability to perceive emotions, discriminate them and using this information to guide thoughts and actions is named empathy, the central feature of the emotional intelligence (Mayer et al., 1990; Lamm et al., 2011). Furthermore, empathy can be understood as an affective and cognitive process of social modulation on emotional responses (Grenier and Luthi, 2010). In this context, the ability to perceive pain has also both clear adaptive and evolutive values (Williams, 2002).

The painful phenomenon has been characterized as a subjective and multidimensional experience (Neugebauer et al., 2009). Its perception and processing result from perceptual, sensory, cognitive, and affective-emotional components (Kim et al., 2014) modulated by the central nervous system in cortical and subcortical structures within pain pathway (Bornhovd et al., 2002; Gebhart, 2004, Suzuki et al., 2004).

This multidimensional pain processing arrangement can be explained by the central network of brain structures that process nociceptive information, i.e., pain pathways. Since cortical structures (more superior), more directly linked with affective-emotional experiences, can modulate the sensorial response of pain through the interaction with more basal structures, such as those of the limbic system. That interaction can be observed, for example, in nocifensive responses. In the same way, experiences and structures more related to the sensorial component of pain influences and modulates experiences and structures more related to the affective-emotional component, such as, for instance, a harmful stimulus that leads to an aversive memory. In both cases, the pain information is being processed in series (Ji et al., 2010; Luongo et al., 2013; Neugebauer, 2015). However, pain inputs can also be processed in parallel, once same-level structures can modulates and work together to generate a specific response to a nociceptive stimulus (Price, 2000).

Although the neurobiological mechanisms which modulate the perceptual and sensory component of pain have been extensively studied, those involved in the affective-emotional and cognitive component are less known (Langford et al., 2006; Borsook and Becerra, 2009; Neugebauer et al., 2009; Shamay-Tsoory, 2011). Recent studies have been demonstrating that the affective-emotional pain component is equally activated in those who are just observing painful or potentially painful situations (Singer et al., 2004; Shamay-Tsoory, 2011). Specific researches using animal models have demonstrated that rodents are able to exhibit one of the major facets of empathy (Zaki and Ochsner, 2012), the pro-social behavior face to a conspecific suffering (Langford et al., 2006, 2010, 2011; Ben-Ami Bartal et al., 2011, 2016; Baptista-de-Souza et al., 2015; Mogil, 2015).

In the light of these findings, researchers have demonstrated that observing a cagemate in pain can increase or decrease pain sensation in the observer when subjected to the writhing, radiant heat paw-withdrawal and the formalin tests (Langford et al., 2006; Ben-Ami Bartal et al., 2016). In this sense, the study conducted by our group has evidenced hypernociception induced by cohabitation with a pair subjected to neuropathic pain (Baptista-de-Souza et al., 2015).

Even though it is not clear which brain structures and neurotransmitters modulate this response (Mogil, 2015), the studies have shown that the reflex face to other experiences results from a specific group of neurons known as mirror neurons, which allows the observer to encode and understand other’s intention by mechanisms of observational learning (Moya-Albiol et al., 2010). This way, through mirror systems, an emotional reaction of an animal may generate a similar emotional representation in the observer, i.e., allowing empathy (Gallese et al., 2004).

The insular cortex is among the cortical encephalic structures involved on pain and empathy processes due to the presence of mirror neurons (Jackson et al., 2006). This structure is involved on modulation of stimulus intensity, and prediction of pain (Kross et al., 2011; Shamay-Tsoory, 2011), as well as being responsible for connections between the sensory cortex and the limbic system (Gasquoine, 2014). Furthermore, neurons located in the insula are responsive to painful stimuli, painful situation clues and even when painful stimuli are applied to another individual (Lamm et al., 2011).

GABA is one of the several neurotransmitter systems located in the insula and it is the main inhibitory neurotransmitter in the mammalian brain (Wiebking et al., 2014). The GABAergic receptors are located in structures that modulate emotional and pain processes (Bushnell et al., 2013; Yowtak et al., 2013; Watson, 2016). For instance, evidences have demonstrated that imbalances – decrease or increase – in GABAergic neurotransmissions and others inhibitory neurotransmitters compared to excitatory neurotransmitters within the insula leads to an altered central pain processing and sensitivity (Watson, 2016).

Recently, there has been an increasing interest to investigate the neurobiological substrate involved in emotional modulation of pain. Based on this body of evidence, we hypothesized that insular cortex, specifically its GABAergic system modulates the hyperalgesia induced by cohabitation with a mouse subjected to sciatic nerve constriction, a neuropathic pain model. Here we performed four experiments to test our hypothesis: (i) intra-insula injections of the cobalt chloride (CoCl_2_) in mice housed in groups, to inactivate the structure; (ii) intra-insula injections of the CoCl_2_ in mice housed in pairs; (iii) systemic injections of midazolam, a GABAA agonist, to investigate the systemic involvement of GABAergic system; and (iv) intra-insula injections of the midazolam, to investigate specific GABAA involvement.

## Materials and Methods

The experiments were performed in compliance with the recommendations of the Brazilian Guidelines for Care and Use of Animals for Scientific and Educational Purposes, elaborated by The National Council of control of animal testing (CONCEA). This study was also approved by the Ethics Committee on Use of Animals of Federal University of São Carlos (Processes 045/2012 and 4752090415).

### Subjects

For this study 194 male Swiss mice at 6–8 weeks of age (Federal University of São Carlos, SP, Brazil) were housed in groups of 10 per cage (cage size: 41 cm × 34 cm × 16 cm) or in pairs (cage size: 30 cm × 19 cm × 13 cm). Animals had free access to food and water in their home cages and were housed in temperature-controlled room (24 ± 1°C). Lights were maintained on a 12-h light/12-h dark cycle (lights on at 7:00 AM), with all behavioral tests carried out during the light phase of the cycle (9:00 AM – 4:00 PM). Different batches of experimentally naive mice were used for experiments.

### Drugs

The following drugs were used: cobalt chloride, 1 mM.100 nL^-1^ (CoCl_2_; Sigma, St. Louis, MO, United States) (Crestani et al., 2010) and midazolam (MDZ) 3.0 and 30 nmol./0.1 μl (intra-insula) and 0.5, 1.0, and 2.0 mg.kg^-1^ (systemic) [(8-chloro-6-(2-fluorophenyl)-1-methyl-4*H*-imidazo (1,5a) (1,4)benzodiazepine), Roche, Brazil] (Nunes-de-Souza et al., 2000; Baptista et al., 2009). CoCl_2_ and MDZ were dissolved in sterile saline (0.9% NaCl).

### Surgery and Microinjection

Bilateral stainless-steel guide cannulae (25-gauge × 7 mm; Insight Instruments, Brazil) was implanted in mice under ketamine and xylazine anesthesia (100 mg.kg^-1^ and 10 mg.kg^-1^, i.p.,) in a stereotaxic frame (Insight Instruments, Brazil) (Baptista et al., 2009). The guide cannula was fixed to the skull with dental acrylic and jeweler’s screws. Stereotaxic coordinates for insula were 0.7 mm anterior to bregma, 3.3 mm lateral and 2 mm ventral to skull surface (Paxinos and Franklin, 2001). A dummy cannulae (33-gauge stainless steel wire; Fishtex^®^, Brazil) inserted into the guide cannulae at the time of the surgery was utilized to reduce the incidence of occlusion. During the surgery animals received ketoprofen (benzene acetic acid, 5 mg.kg^-1^) and ceftriaxone (ceftriaxone sodium hemipentahydrate, 4 mg.kg^-1^). Before tests mice remained for 4 to 5 days to recover from the surgery (Würbel, 2011).

Solutions were injected into structures by a microinjection unit (33-gauge stainless steel cannula, Insight Instruments, Brazil), that extended 2 mm beyond the tip of the guide cannula. The microinjection unit was connected to a 10 μl Hamilton microseringe via polyethylene tube (PE-10) and the rate of flow was controlled by an infusion pump (BI 2000 – Insight Instruments, Brazil), programmed to deliver 0.1 μl of each solution over a period of 60 s. The microinjection procedure consisted of gently restraining the mice, inserting the injection unit, infusing the solution for 60 s and keeping the injection unit in place for 90 s. The movement of a small air bubble in the PE-10 tube, during and after the microinjection, confirmed the delivery of the solution.

### Sciatic Nerve Constriction

Bennett and Xie (1988) and Sommer (1999) method was used to reproduce the neuropathic pain model. After ketamine and xylazine anesthesia (100 mg.kg^-1^ and 10 mg.kg^-1^, respectively, i.p.,), the fascia between the gluteus and biceps femoral is sectioned and the right sciatic nerve is exposed close to its trifurcation (Sommer, 1999). The tissue around the nerve was carefully cut at approximately 8 mm distance and then the nerve was compressed with three ligatures using sterile non-inflamatory mononylon 6.0 threads (Bennett and Xie, 1988; Sommer, 1999).

### Nociception Test

Nociception was assessed by the writhing test, previously describe by Vander Wende and Margolin (Van der Wende and Margolin, 1956). Among animal models used to evaluate pain mechanisms (Whiteside et al., 2008), the writhing test consists of the application of irritant substances in the peritoneal cavity of rodents, allowing the measure of pain by recording the number of writhes induced by this chemical stimulus. This stimulus induces a visceral acute tonic and diffuse painful sensation mediated by spinal and supra spinal sites, evoking different emotional responses (Tanimoto et al., 2003). In the present study, writhes were induced by injection of 0.1 ml.10g^-1^ b.w. 0.6% acetic acid i.p., 10 min after intra-cerebral CoCl_2_ and MDZ injection and 30 min after systemic MDZ injection (Nunes-de-Souza et al., 2000).

### General Procedure

After 14 days, the mice were divided into two groups: cagemate nerve constriction (CNC), in which one animal of each pair was subjected to sciatic nerve constriction; cagemate sham (CS), in which one animal from each pair was subjected to the same surgery but without constriction. After an additional 14 days of cohabiting, the cagemates (not the mice subjected to the sciatic nerve constriction or sham) were evaluated.

After the treatments (intra-insula or systemically) the mice housed in group (Experiment 1) or cagemates (Experiments 2, 3, and 4) were placed in a cage for 5 min, during which the number of writhes was recorded. All sessions were recorded with a camera linked to a computer in an adjacent laboratory room and data were subsequently evaluated using the program X-Plo-Rat 2005 1.1.0 (Garcia et al., 2005).

Some animals underwent to sciatic nerve constriction were evaluated for the effectiveness of the surgical procedure. For this evaluation, animals underwent to the hot plate test in which latency of paw withdrawal was measured. Each mouse was placed on a metal surface maintained at 52.0 ± 0.2°C. The latency to respond to the heat stimulus was measured using a hand controlled timer. Mice remained on the plate until they performed hind paw lick or hind paw shake, which are considered typical nociceptive responses. Animals were removed from the plate immediately after responding or after a maximum of 60 s (cut off) to avoid tissue damage (Table 1; Baptista-de-Souza et al., 2015).

**Table 1:** Effect on mechanical hypersensitivity observed after 14 days of sciatic nerve constriction.

HOT PLATE TEST	

Groups	Paw withdrawal latency (s)
SHAM	23.55 ± 0.62
CNC	11.91 ± 0.73^∗^


*Responses are presented as Mean ± SEM (*n* = 75). ^∗^*p* < 0.05 vs. Sham group.*

#### Experiment 1: Evaluation of Insular Modulatory Role in Nociception

For this experiment at 21 days post-birth (weaning) mice were housed in groups of 10 per cage. Twenty to twenty-five days after the beginning of cohabiting the subjects underwent stereotaxic surgery. Four to five days after stereotaxic surgery, animals received intra-insula saline or CoCl_2_ microinjections, forming two groups: saline (animals without insula inactivation); and CoCl_2_ (animals with insula inactivation). 10 min later they were i.p., injected with 0.6% acetic acid (0.1 ml/10g b.w.), the nociceptive stimulus. The number of writhes during a 5-min period was recorded. Animals that do not writhing at 5-min period were excluded from the experiment.

#### Experiment 2: Evaluation of Insular Modulatory Role on Social Modulation of Pain and Nociception in Animals That Cohabited in Pairs With Mice Subjected to Sciatic Nerve Constriction

**FIGURE 1:**
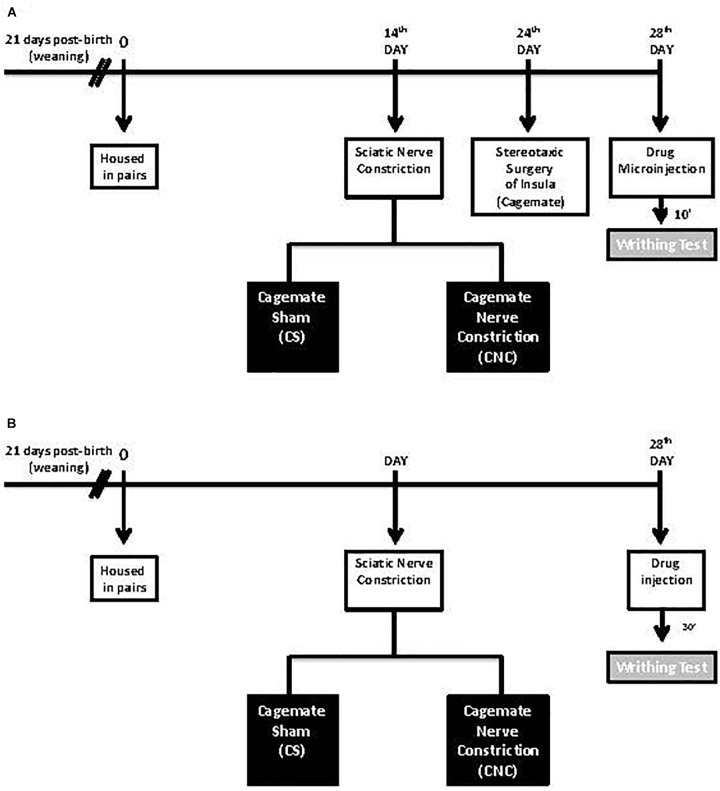
**(A,B)** Schematic representation of the experimental protocol.

At 21 days post-birth, mice were housed in pairs. 14 days after the beginning of cohabiting, they were divided into two groups: CNC, in which one animal of each pair was subjected to sciatic nerve constriction, as described above; and, CS in which one animal from each pair was subjected to the same surgery as CNC group but without nerve constriction. On the 24th day cagemates underwent stereotaxic surgery. On the 28th day, cagemates were subjected to the same after-stereotaxic surgery procedures described in Experiment 1 (Figure 1A).

#### Experiment 3: Evaluation of the Role of Systemic Midazolam on Nociception in Mice After Cohabited With a Pair in Chronic Pain Condition Induced by Sciatic Nerve Constriction

For this experiment, it was utilized the same protocol as described in Experiment 2, except that cagemate animals were not submitted to stereotaxic surgery. In the 28th day, the cagemates received subcutaneous midazolam or saline injection and, 30 min after, were subjected to writhing test as the procedure described in Experiment 1 (Figure 1B).

#### Experiment 4: Evaluation of the Role of Midazolam Intra-Insula on Nociception in Mice After Cohabited With a Pair in Chronic Pain Condition Induced by Sciatic Nerve Constriction

Animals were submitted to the same protocol as described in Experiment 2, excepted that in the 28th day cagemates received intra-insula saline or midazolam microinjections and, 10 min after, were subjected to writhing test described in Experiment 1 (Figure 1A).

### Histology

At the end of the tests, all animals of experiments 1, 2, and 4 received a 0.1 μl infusion of 2% Evans blue, according to the microinjection procedure described above. After ketamine and xylazine anesthesia (100 mg.kg and 10 mg.kg, i.p.,) animals were killed by cervical dislocation, their brains were removed, and injection sites verified histologically according to the atlas of Paxinos and Franklin (2001). Data from animals with injection sites outside the required structure were excluded from the study.

### Statistical Analysis

After Levene’s test to confirm the data homogeneity, in Experiment 1 data were analyzed by using Student’s *t*-test for independent samples. In experiment 2, 3, and 4 data were analyzed by using analysis of variance (ANOVA) of two factors (factor 1: treatment; and factor 2: cohabitating type). Cases of significance were further analyzed by Duncan’s multiple comparison tests. A *p*-value of 0.05 or less was required for significance.

## Results

### Hot Plate: Measurement of Surgical Procedure Effectiveness in Animals Subjected to Sciatic Nerve Constriction

Results of the hot plate test revealed statistically significant effects (*t_38_* = 12.10, *p* < 0.05). The *post hoc* Duncan test indicated a decrease of response latency to the heat stimulus in mice subject to the sciatic nerve constriction compared to sham animals (Table 1).

**FIGURE 2:**
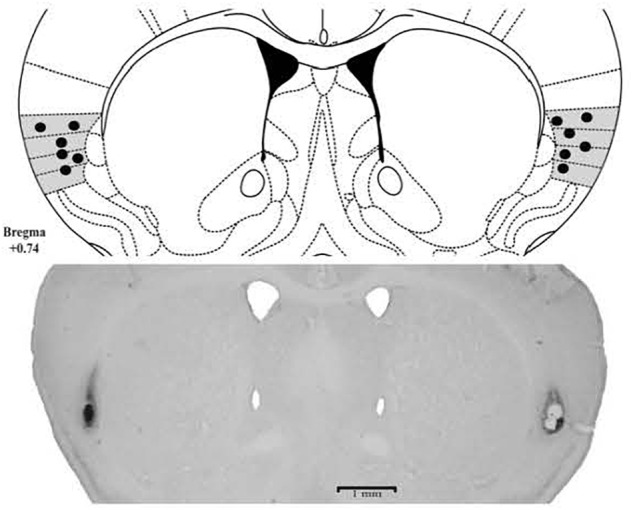
Photomicrographs and schematic representation of histological results according to the Atlas of Paxinos and Franklin (2001) in insula. The filled circles represent the sites of drug infusion.

The histological analysis confirmed that 103 mice received bilateral cannula implantation in the Insula [Experiment 1: Saline (*n* = 9), CoCl_2_ (*n* = 8); Experiment 2: CS/Saline (*n* = 7), CS/CoCl_2_ (*n* = 7), CNC/Saline (*n* = 9), CNC/CoCl_2_ (*n* = 7); Experiment 4: CS/SALINE (*n* = 9), CS/MDZ 3 (*n* = 8), CS/MDZ 30 (*n* = 10), CNC/SALINE (*n* = 10), CNC/MDZ 3 (*n* = 9), CNC/MDZ 30 (*n* = 10)] (Figure 2).

### Experiment 1: Evaluation of Insular Modulatory Role in Nociception

**FIGURE 3:**
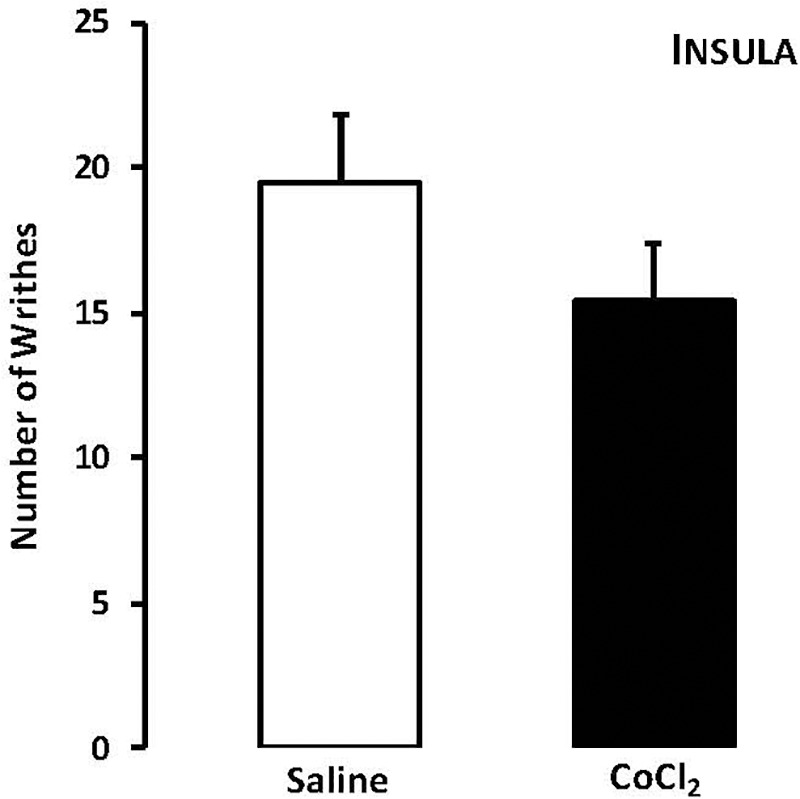
Effects of microinjection in insula (*n* = 17) of saline or CoCl_2_ (1 mM/0.1 μl) on number of writhing in mice housed in groups. Data are presented as mean ± SEM.

For mice that lives in groups and received intra-insula injections of saline or CoCl_2_, Student’s *t*-test revealed no statistically significant effects for the type of treatment factor (*t*_15_ = -0.98, *p* > 0.05) (Figure 3).

### Experiment 2: Evaluation of Insular Modulatory Role on Social Modulation of Pain and Nociception in Animals That Cohabited in Pairs With Mice Subjected to Sciatic Nerve Constriction

For mice that cohabited in dyads, two-way ANOVA revealed statistically significant effects for cohabitation factor [*F*_(1,26)_ = 19.52, *p* < 0.05]. Duncan’s test revealed that, despite of treatment, cohabitating with animals underwent to chronic constriction injury promotes an increased in number of writhes in cagemates when compared with respective group sham.

**FIGURE 4:**
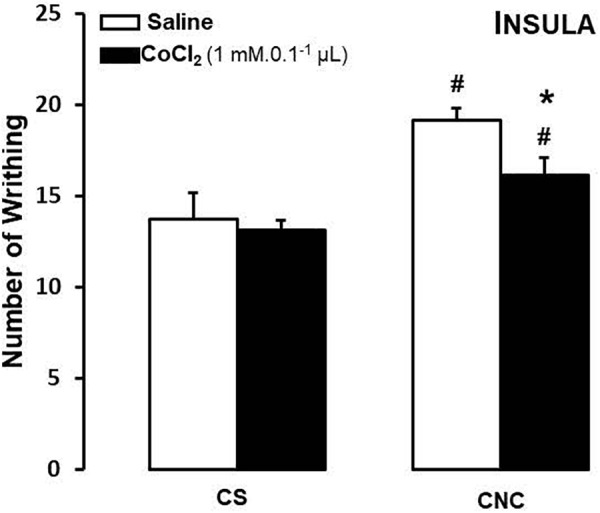
Effects of microinjection in insula (*n* = 30) of saline or CoCl_2_ (1 mM/0.1 μl) on number of writhing in mice housed in pairs. Data are presented as mean ± SEM. ^∗^*p* < 0.05 vs. respective saline group. #*p* < 0.05 vs. respective CS group. CNC, cagemate nerve constriction; CS, cagemate sham.

Although statistical analysis showed a non-effect for treatment factor [*F*_(1,26)_ = 3.47; *p* = 0.07] neither to treatment and cohabitation factors interaction [*F*_(1,26)_ = 1.59; *p* > 0.05], ANOVA have been shown a *p*-value very close to significance. Therefore, Duncan’s test revealed a decrease in number of writhes only in CoCl_2_-treated/CNC group when compared with saline-treated/CNC animals (Figure 4).

### Experiment 3: Evaluation of Systemic Midazolam Treatment on Nociception in Mice After Cohabited With a Pair in Chronic Pain Condition by Sciatic Nerve Constriction

**FIGURE 5:**
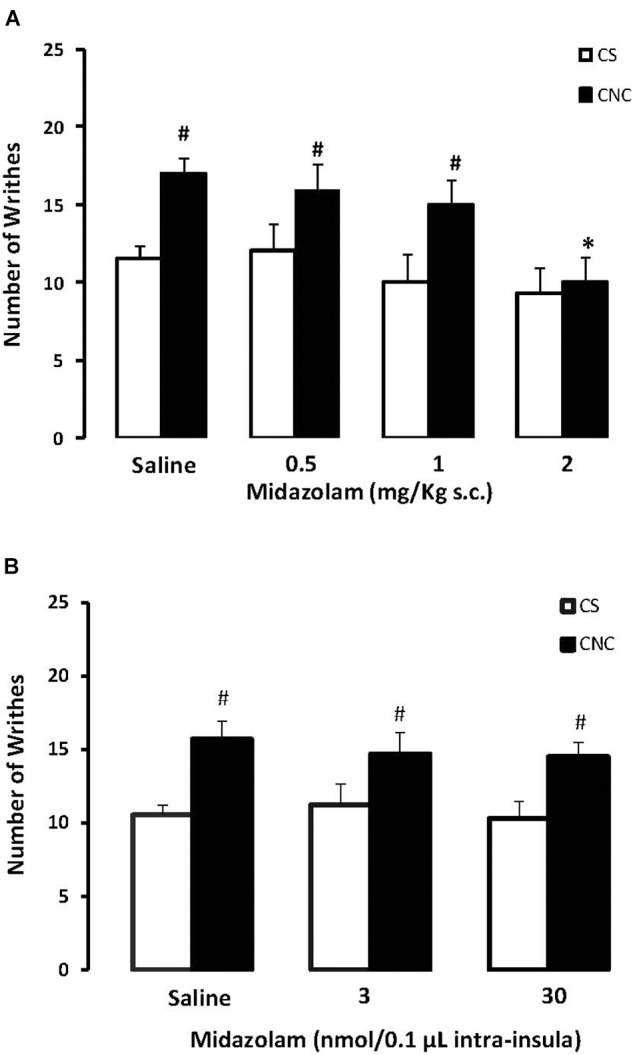
(**A**) Effects of midazolam (0.5, 1.0, and 2.0 mg/kg, s.c.) or saline systemic injections (*n* = 89) on number of writhing in mice housed in dyads. Data are presented as mean ± SEM. ^∗^*p* < 0.05 vs. respective saline group. #*p* < 0.05 vs. respective CS group. CNC, cagemate nerve constriction; CS, cagemate sham. (**B**) Effects of microinjection in insula (*n* = 57) of midazolam (3 and 30 nmol/0.1 μl) or saline on number of writhing in mice housed in dyads. Data are presented as mean ± SEM. #*p* < 0.05 vs. respective CS group. CNC, cagemate nerve constriction; CS, cagemate sham.

Two-way ANOVA revealed statistically significant effects for cohabitation factor [*F*_(1,82)_ = 13.38; *p* < 0.05] and for treatment factor [*F*_(3,82)_ = 4.10; *p* < 0.05]. Duncan’s test revealed an increase of writhes in cagemates that cohabitating with animals subjected to chronic constriction injury when compared with animals that cohabited with a sham animal. *Post hoc* test also revealed that the higher dose of (MDZ) (2.0 mg.kg) decreased the number of writhes in CNC animals compared to respective saline group. None of the doses of midazolam interferes with the number of writhes in CS animals, compared to respective saline group (Figure 5A).

### Experiment 4: Evaluation of Midazolam Intra-Insula Treatment on Nociception in Mice After Cohabited With a Pair in Chronic Pain Condition Induced by Sciatic Nerve Constriction

For mice that received intra-insula injections of MDZ two-way ANOVA followed by *post hoc* Duncan’s test demonstrate statistically significant effects for cohabitation factor [*F*_(1,39)_ = 10.32; *p* < 0.05] but no effects for treatment factor [*F*_(2,39)_ = 0.17; *p* > 0.05] compared to respective saline group. Duncan’s test revealed an increase of writhes in cagemates that cohabitating with animals subjected to chronic constriction injury when compared with animals that cohabited with a sham animal (Figure 5B).

## Discussion

The present findings corroborate with previous studies where the cohabitation with a cagemate in chronic pain condition was able to promote hyperalgesia (Baptista-de-Souza et al., 2015). Interestingly, the insula inactivation, induced by CoCl_2_ attenuated the nociceptive response only in animals that cohabited with a cagemate in chronic pain condition, but on the other hand did not alter the number of writhes in mice housed in groups. Nevertheless, systemic treatment with midazolam (2.0 mg.kg^-1^) decreased the hyperalgesia induced by living with a pair undergone through sciatic nerve constriction. However, we found that activation of GABAA receptors in the insula did not change this hypernociceptive effect induced by conspecific submitted to neuropathic pain model.

Several findings have been demonstrated that rodents showed emotional reactions (Mony et al., 2018) face to the conspecific suffering pain, and that cagemate condition is able to influence their pain sensitivity (Langford et al., 2006, 2011). Recently, our group has shown an anxiogenic-like effect by analyzing behavioral changes in anxiety parameters in mice tested on elevated plus-maze and open field tests after coexisting with a conspecific submitted to sciatic nerve constriction (Baptista-de-Souza et al., 2015), which is an evidence that strengthens the significance of social factors and their affecting role on pain sensitivity (Mogil, 2017).

On the other hand, previous studies have considered chronic pain as a stressful situation (Ulrich-Lai et al., 2006; Vachon-Presseau et al., 2013), and it could alter nociception in different ways (attenuation or increase responses) (Coutinho et al., 2002; Tramullas et al., 2012). Furthermore, it has been demonstrated that induction of stress reverted emotional contagion of pain in mice (Martin et al., 2015). Concerning these evidences, Baptista-de-Souza et al. (2015), specifically in the protocol used in our study, did not observe differences on corticosterone levels in mice that cohabited 14 days with a cagemate in chronic pain condition, suggesting that this effect is not related to stress, but to an emotional contagion, i.e., an evolutionary behavior precursor of empathy in mammals (Preston and de Waal, 2002).

Moreover Langford et al. (2006), have shown that the observation of a conspecific in pain allow sensitization in pain pathways, inducing the called “state of priming” in the brain. In this way, a nociceptive stimulus applied after a certain previous emotional situation (priming) leads to the exacerbation of the subsequent painful experience in cagemates, but not in strangers (Langford et al., 2006; Ben-Ami Bartal et al., 2011). In brief, we suggested that cohabitation with an animal in suffering (emotional situation – priming) was able to promote the sensitization of pain neural circuits in the cagemate, and consequently, when the animal was submitted to a painful situation, the nociceptive sensation would be exacerbated.

Among the encephalic areas that could modulate these responses, the insula has a significant role in painful or potentially painful situations and on social modulation of pain (Peyron et al., 2000; Decety et al., 2016). To confirm the evidence cited above, our second experiment demonstrated that insula inactivation produced a decrease on abdominal writhes number in CNC but not in CS group.

The insular cortex exhibits an extensive and multifaceted connectivity during painful situations (Saper, 1982; Starr et al., 2009). Studies have demonstrated that activation of insula could produce antinociceptive and pronociceptive effects (Derbyshire et al., 2004; Craggs et al., 2007). Concerning this, patients with insula lesions can present complex altered behavioral in painful situations, as asymbolia, but without affecting the nociceptive threshold (Berthier et al., 1988).

Also regard the role of insular cortex in the modulation of pain, Langford et al. (2010) have demonstrated that insula lesions in mice were able to attenuate facial expressions of pain without affecting the number of writhes, evidencing that the inactivation of this structure in an acute pain situation alters the emotional component of pain significance, but not the sensorial behavior component-related to pain. In our work, the insula inactivation decreased the number of abdominal writhes, attenuating the hyperalgesia only in a cagemate that cohabited with a mouse subjected to sciatic constriction, an emotional situation, without altering the nociceptive response in mice housed in groups.

The first experiment was performed in order to investigate the involvement of insular cortex in the pain response *per se* (without emotional influence). We considered that is an important previous evaluation to better featuring the role of this cortical area in the emotional component of pain. However, we observed that the inactivation of insula in animals that lived in groups (normal experimental situation) (Experiment 1) did not alter the pain response. Therefore, we have been showed the insula inactivation, induced by CoCl_2_ attenuated the nociceptive response only in animals that cohabited with a cagemate in chronic pain condition.

Although we considered the control situation is the animal living with a SHAM cagemates, this type of cohabitation (in pairs) is different of those commonly applied in laboratory environmental, wherein the animals living in groups. Curiously, the number of writhes in mice treated with saline and that living in groups was superior compared with those in the same condition but that lived in pairs. This fact leads to consider that the pattern of writhing in both control situations (saline treatment) can be explained by housing the animals in pairs or groups.

Mice are social animals and the isolation can be responsible for behavior alterations, including pain sensitization (Puglisi-Allegra and Oliverio, 1983). Suchlike a social isolation, we believe that the housing with small groups or pairs can promote a bigger state of vigilance when compared with animals that cohabitate in larger groups. According to aversive brain system model (Gray and McNaughton, 2000), this major vigilance threshold leads to non-defensives behavior inhibition, like those related to pain. Therefore, although the writhes average of the control group that lived in pairs show up smaller than the average in the same group of the animals that lived in groups; they are similar to those observed in the experiments of our groups (Baptista-de-Souza et al., 2015). This fact leads us to propose the hypothesis that the pain threshold is related to the cohabitation type.

Although the neurochemical substrate involved in empathy for pain is not yet established (Mogil, 2015), subjects in chronic pain conditions display neurotransmitter system imbalances in some encephalic structures (Bushnell et al., 2013; Watson, 2016) as well as comorbidities with emotional disturbances (Gamsa, 1990; Edwards et al., 2016).

In this context, the experiments 3 and 4 investigated the involvement of the GABAA-benzodiazepine receptors system in the social modulation of pain through systemic or intra-insula midazolam microinjections. We observed that a higher dose of systemic midazolam reversed hyperalgesia induced by cohabitation in the CNC group, demonstrating a possible involvement of the neurotransmitter system on emotional process.

Although the analgesic effects of midazolam have already been reported by the literature (Rodgers and Randall, 1987; Niv et al., 1988; Nishiyama et al., 1999), specifically in the writhing test (Nunes-de-Souza et al., 2000), in the present study the analgesic effect was observed only by the highest dose in dyads, i.e., demonstrating its effects only on the emotional component of nociception.

Furthermore, Ben-Ami Bartal et al. (2016) have been reported that midazolam impaired helping behavior in rats, one of the faces of empathy, whereby withdrawals the emotional aspect of experimental condition and this effect is not due to the indirect sympatholytic effect. Considering above mentioned findings, and ours results obtained from Experiments 2 and 3, we injected intra-insula midazolam. However, curiously we did not observe changes in the hypernociception induced by cohabitation with a mouse in neuropathic pain condition.

Concerning that, it has been described that inactivation by cobalt chloride (as conducted in Experiments 1 and 2) blocked several active neurotransmitters within the structure (Kretz, 1984; Lomber, 1999). Thus, the effects of insular GABAergic system may not have occurred likewise in Experiment 2 due the coordinated participation of this inhibitor neurotransmitter with others neurotransmitters (Watson, 2016) in this social modulation of pain condition.

In addition, dopamine and serotonin have been described as important neurotransmitters on emotional processes, nociception and pathology related to empathy, as well, schizophrenia and autism (Elhwuegi, 2004; Nakamura et al., 2010; Komuniecki et al., 2012; Flood and Clark, 2014). In the same way, the study performed by Watson (2016), demonstrated that the unbalanced excitatory and inhibitory neurotransmitters within insula could increase thermal hyperalgesia and mechanical allodynia in subjects in chronic pain conditions.

Likewise the insular cortex, the emotional response and pain are also processed in the amygdaloid complex (Fields, 1999; LeDoux, 2000). It has been reported that GABAA receptors within amygdala processes sensory and affective pain in rats under neuropathic pain condition (Pedersen et al., 2007). In addition, previous results of our group had demonstrated alterations in nociception after inactivation of the amygdala in mice that lived with a cagemate submitted to a neuropathic model (Pelarin et al., unpublished).

Taken together, the present study has demonstrated that (i) cohabitation in pairs with a cagemate in a chronic pain condition induces hypernociception, (ii) insular cortex is one of the neural substrate of empathy for pain, (iii) GABAA-benzodiazepine receptors system is involves on the modulation of hyperalgesia induced by living with a conspecific in neuropathic pain condition, and (iv) the activation of GABAergic neurotransmission within insula did not interfere in the cagemates nociceptive responses housed with a pair in chronic pain suffering.

## Conclusion

This work has performed in order to answer two hypotheses; the hyperalgesia induced by cohabitation with a mouse in chronic pain condition is modulates by insular cortex (confirmed wherein the inactivation of the insula blocked this effect). Furthermore, this modulation of insular cortex would occur by GABAergic system action, which was not confirmed whereas the midazolam injections in the insula did not change the pain responses (Experiment 4).

## Author Contributions

CZ and VP contributed in carrying out the experiments, data analysis, and drafting the manuscript. DB-d-S contributed in the formulation and supervision of the experiments, and drafting the manuscript. AC-d-S conceived the study, gave instructions on experimental design, and performed the manuscript writing supervision.

## Conflict of Interest Statement

The authors declare that the research was conducted in the absence of any commercial or financial relationships that could be construed as a potential conflict of interest.
